# Supplementary material for: TAS3351 is a brain penetrable EGFR-TKI that overcomes T790M and C797S resistant mutations
Source: Commun Med (Lond). 2026 Mar 25;6:284. doi: 10.1038/s43856-026-01546-1 (PMC13181001; doi:10.1038/s43856-026-01546-1)
Supplement: Supplementary file 2 — Supplementary Information [file 43856_2026_1546_MOESM2_ESM.pdf]

Supplementary Information

Supplementary methods

Surface plasmon resonance (SPR) analysis

All biotinylated recombinant EGFR proteins were purchased from Carna Biosciences. 5 µg of biotinylated EGFR proteins were immobilized in Series S Sensor Chip SA (Cytiva) with PBS-P+ buffer (Cytiva) and 5 mmol/L $MgCl_2$ at 22°C using Biacore T200 (General Electric Company). All tested compounds were dissolved in DMSO and added to running buffer (PBS-P+, 5 mmol/L $MgCl_2$) adjusted to 1% DMSO. Affinity to recombinant EGFR proteins was calculated via single-cycle kinetics using Biacore Evaluation Software (version 3.0). Obtained sensorgrams were subjected to curve fitting using 1:1 binding model and calculated K_D values. When the affinity of the test compound for the recombinant EGFR was insufficient for curve fitting, a steady-state affinity evaluation was performed.

Supplementary Fig 1. Chemical structure of EGFR-TKIs in the series of pyrrolopyrimidine-quinoline chemical core structure. The chemical structures of TAS3351 (a), TAS3351 analog, Compound 1 (b), TAS-121 (c), and zipalertinib/TAS6417/CLN-081 (d), those are all EGFR-TKIs sharing the pyrrolopyrimidine-quinoline chemical core structure. Red-highlighted parts of the chemical structures indicate the pyrrolopyrimidine-quinoline.

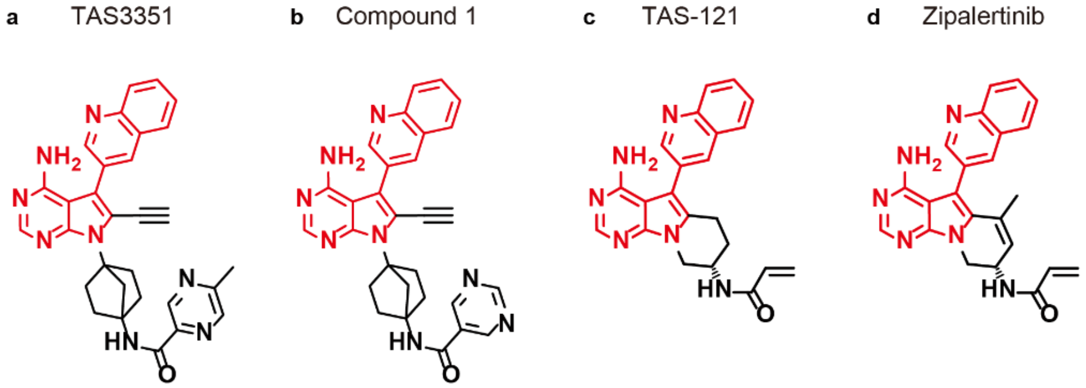

Supplementary Fig 2. Mean IC_{50} values of *in vitro* enzymatic inhibition in TAS3351, erlotinib, and osimertinib in ATP concentrations relevant to K_m values in each recombinant EGFR. Enzymatic inhibition was measured via Off-chip Mobility Shift Assay (MSA) using recombinant wild-type and mutated EGFR in the ATP concentration relevant to K_m values of each human recombinant EGFR. ATP concentrations in the assay in each recombinant EGFR are as follows: wild type, ex19del/T790M, and L858R/ C797S, 5 µmol/L; ex19del, 25 µmol/L; ex19del/C797S, and L858R, 10 µmol/L; ex19del/T790M/C797S, and L858R/T790M, 2 µmol/L. Bars indicate the mean IC_{50} value and error bars indicate standard deviation (s.d.) from triplicates. The individual IC_{50} values are shown by dots.

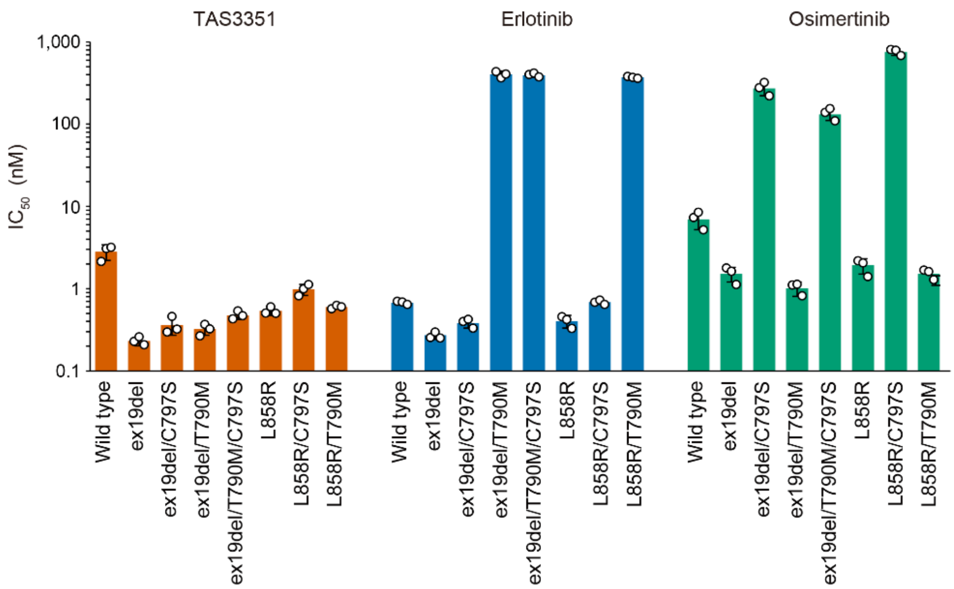

Supplementary Fig 3. The K_D values of TAS3351 and erlotinib in human recombinant EGFR. The affinity of TAS3351 and erlotinib to biotinylated recombinant human EGFR was evaluated using surface plasmon resonance (SPR) analysis with single-cycle kinetics. K_D values were calculated via curve fitting with 1:1 binding model or steady-state affinity evaluation. Mean K_D values and standard deviation (s.d.) obtained from three independent experiments are shown.

EGFR genotype	K_D (nM, mean ± SD)	
	TAS3351	Erlotinib
Wild type	4.78 ± 0.477	0.932 ± 0.140
ex19del	1.01 ± 0.318	0.558 ± 0.461
ex19del/C797S	0.995 ± 0.345	0.661 ± 0.412
ex19del/T790M	4.67 ± 1.52	6.19 ± 3.51
ex19del/T790M/C797S	0.420 ± 0.0308	27.1 ± 13.3
L858R	2.98 ± 0.283	0.770 ± 0.0153
L858R/C797S	3.32 ± 3.09	2.21 ± 1.31
L858R/T790M	1.85 ± 0.478	33.5 ± 16.8
L858R/T790M/C797S	0.448 ± 0.158	72.9 ± 6.84
T790M	4.41 ± 2.18	19.2 ± 11.9

Supplementary Fig 4. Kinome-wide selectivity of TAS3351. The inhibitory potency of 20 nmol/L TAS3351 among 255 kinases was evaluated with Off-chip Mobility Shift Assay. Twenty-six kinases demonstrated that over 50% of kinase reactions were inhibited by 20 nmol/L of TAS3351. The inhibitory potency with the titrated concentration of TAS3351 was evaluated in the 26 kinases, followed by the determination of the IC_{50} values. Eight of these kinases showed the IC_{50} values below that in wild-type EGFR.

Kinase	% inhibition	IC_{50} (nmol/L)
EGFR (ex19del)	ND	0.339
HER4	94.7	0.594
EPHA5	96.0	1.13
EPHB4	97.7	1.54
EPHB3	92.2	1.70
EPHB2	95.3	2.52
EPHA4	94.2	2.63
RET	89.8	2.83
YES	84.8	3.54
EGFR	85.8	3.56
EPHA1	83.8	3.70
EPHA6	96.6	4.25
EPHA8	75.6	6.23
DDR1	79.5	6.40

Kinase	% inhibition	IC_{50} (nmol/L)
BRK	74.7	6.82
PDGFRα	72.6	8.73
EPHB1	70.7	9.03
TXK	66.6	11.4
DDR2	70.9	11.5
FLT4	67.5	12.7
EPHA3	62.8	12.8
CK1δ	57.2	13.6
CK1ε	67.0	15.6
EPHA7	51.8	18.9
HER2	56.0	19.7
FRK	55.9	20.9
EPHA2	62.8	23.5

Supplementary Fig 5. Body weight evaluation by oral administration of TAS3351 and reference EGFR-TKIs in mouse models bearing allografts of NIH/3T3-EGFR cells and xenografts of human cancer cell lines. a-d, the body weight evaluation by oral administration of TAS3351, erlotinib, and osimertinib was evaluated in nude mice (BALB/cAJcl-*nu/nu*) subcutaneously implanted with murine NIH/3T3 embryonic fibroblast cells stably expressing human EGFR with the following mutations: ex19del/C797S (a), L858R/C797S (b), ex19del/T790M/C797S (c), and L858R/T790M/C797S (d). **e-i,** the body weight evaluation by oral administration of TAS3351 and reference EGFR-TKIs was evaluated in mice subcutaneously bearing xenografts of human cancer cell lines. The EGFR expressed in the respective human cancer cell lines is as follows: HCC827 (ex19del, **e**), PC-9 (ex19del, **f**), PC-9 (C797S) (ex19del/C797S, **g**), NCI-H1975 (L858R/T790M, **h**), and A-431 (wild-type, **i**,). Number of mice in each group was five, except for PC-9 (C797S), in which the number was six, and unless otherwise specified. Based on the predefined humane endpoint criteria for this study, † each animal in vehicle control and erlotinib group was euthanized under anesthesia on day 11 and day 5 respectively due to ulceration in tumors, and ‡ an animal in vehicle control and two animals in TAS3351 50 mg/kg group were euthanized under anesthesia on day 8. The mouse in the 80 mg/kg TAS3351 group in HCC827, and the mouse in the vehicle control and 20 mg/kg erlotinib group in PC-9 were excluded from the evaluation, based on the predefined exclusion criteria (administration error). The mean of the relative body weight change is plotted, and error bars represent the standard error of the mean (s.e.m.). All compounds were administered orally once daily (QD) starting from day 1. In the control group, vehicle (20% HP-β-CD, 0.1 mol/L HCl) was administered as a control for TAS3351. This body weight evaluation was concurrently performed with the tumor volume evaluation showed in Fig. 5a-i.

Supplementary Fig 5.

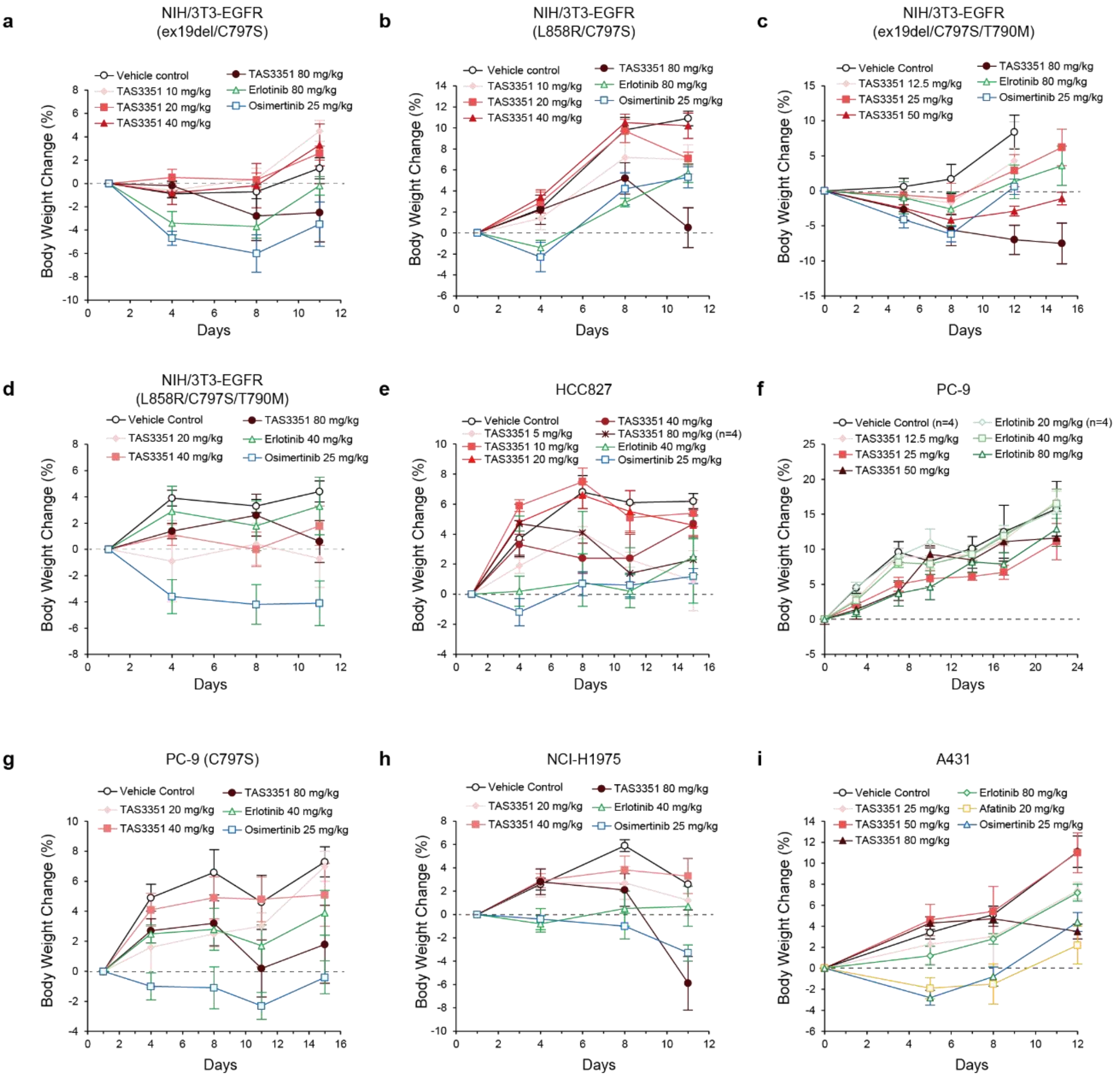

Supplementary Fig 6. Uncropped immunoblotting images for Fig 6a.

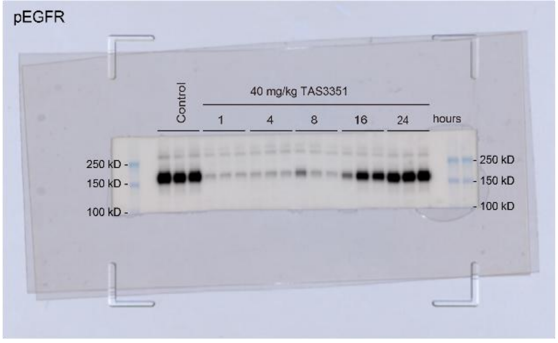

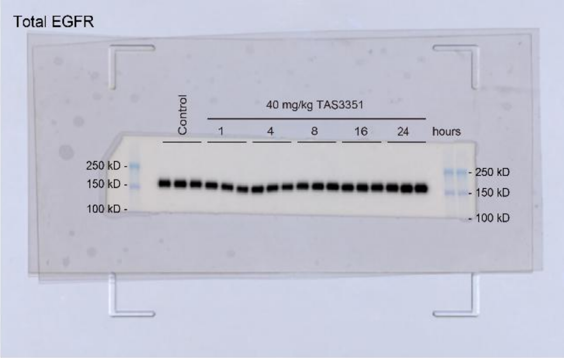

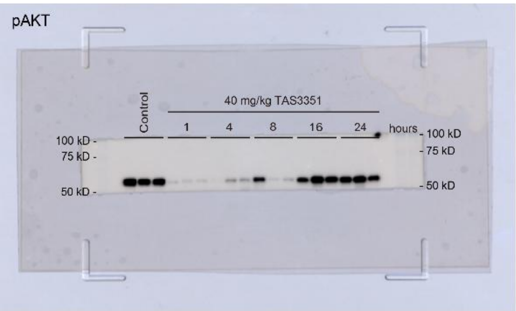

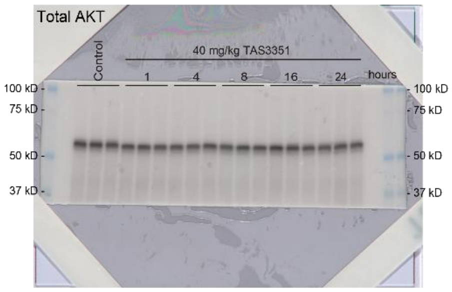

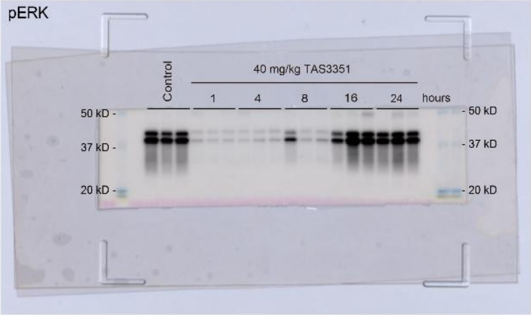

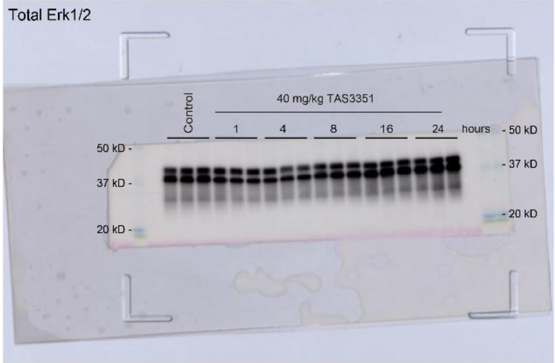

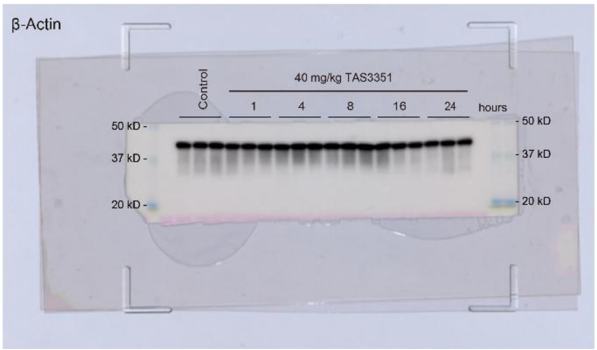

Supplementary Fig 7. Uncropped immunoblotting images for Fig 6b.

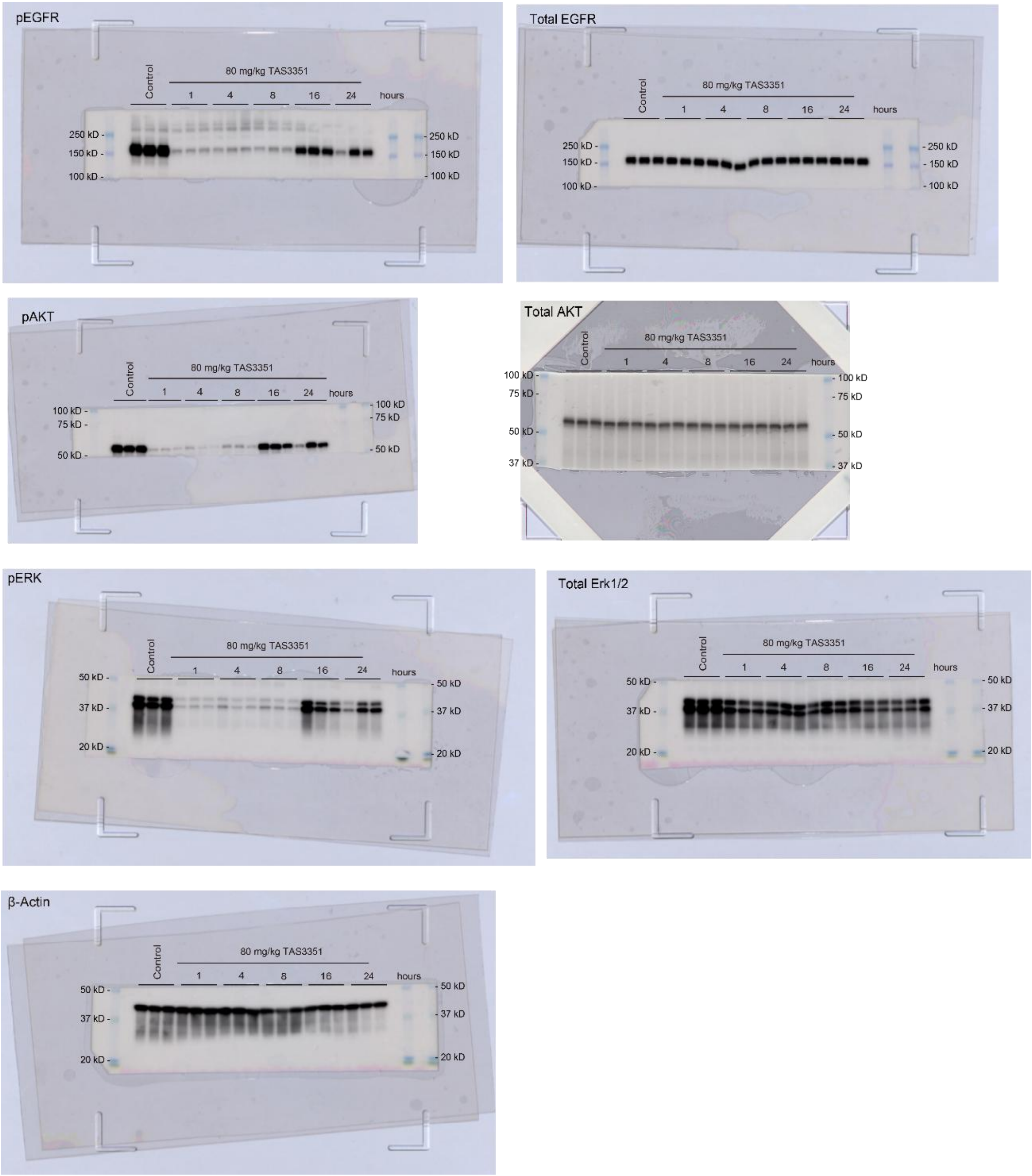

Supplementary Table. 1. Data collection and processing statistics

**PDB ID**	**9KL4**	**9KLW**
**Protein**	**EGFR (wild type)**	**EGFR (T790M/L858R)**
**Ligand**	**Compound 1**	**Compound 1**
X-ray source	PXII/X10SA (SLS[a])	PXII/X10SA (SLS[a])
Wavelength [Å]	0.9998	1.0000
Detector	EIGER	EIGER
Temperature [K]	100	100
Space group	I 2 3	I 2 3
Cell: a; b; c; [Å]	146.92; 146.92; 146.92	145.99; 145.99; 145.99
α; β; γ [°]	90.0; 90.0; 90.0	90.0; 90.0; 90.0
Resolution [Å]	2.33 (2.37–2.33)	2.67 (2.71–2.67)
Unique reflections	22851 (1163)	14821 (750)
Multiplicity	24.7 (25.6)	24.6 (25.2)
Completeness [%]	100.0 (100.0)	99.3 (100.0)
R_{sym} [%][b]	8.7 (285.4)	12.0 (289.1)
R_{meas} [%][c]	8.9 (291.2)	12.3 (295.0)
Mean(I)/sd[d]	21.8 (1.3)	20.2 (1.3)

[a]Swiss Light Sourse (SLS, Villigen, Switzerland)

[b]$R_{sym} = \sum_h \sum_i^{n_h} \left|\widehat{I_h} - I_{h,i}\right| / \sum_h \sum_i^{n_h} I_{h,i}$ with $\widehat{I_h} = \frac{1}{n_h} \sum_i^{n_h} I_{h,i}$

where $I_{h,i}$ is the intensity value of the ith measurement of h

[c]$R_{meas} = \sum_h \sqrt{\frac{n_h}{n_h - 1}} \sum_i^{n_h} \left|\widehat{I_h} - I_{h,i}\right| / \sum_h \sum_i^{n_h} I_{h,i}$ with $\widehat{I_h} = \frac{1}{n_h} \sum_i^{n_h} I_{h,i}$

where $I_{h,i}$ is the intensity value of the ith measurement of h

[d]calculated from independent reflections

Supplementary Table 2. Refinement statistics[a].

**PDB ID**	**9KL4**	**9KLW**
**Protein**	**EGFR (wild type)**	**EGFR (T790M/L858R)**
**Ligand**	**Compound 1**	**Compound 1**
Resolution [Å]	103.89–2.32	103.23–2.67
No. of reflections (working /test)	21538 / 1296	13988 / 833
R_{cryst} [%]	18.6	19.7
R_{free} [%][b]	23.8	25.7
Total number of atoms:		
Protein	2504	2401
Water	54	15
Ligand / Chloride / DMSO	38 / 1 / 0	38 / 0 / 4
Deviation from ideal geometry:[c]		
Bond lengths [Å]	0.012	0.011
Bond angles [°]	1.58	1.43
Bonded B's [$Å^2$][d]	13.6	5.6
Ramachandran plot:[e]		
Most favored [%]	89.5	93.1
Additional allowed [%]	9.4	6.9
Generously allowed [%]	0.4	0.0
Disallowed [%]	0.7	0.0

[a]Values defined in REFMAC5, without a sigma cutoff. [b]Test-set contains 4.4% of the measured reflections. [c]Root mean square deviations from geometric target values. [d]Calculated using MOLEMAN. [e]Calculated using PROCHECK.
