# Supplementary material for: TAS3351 is a brain penetrable EGFR-TKI that overcomes T790M and C797S resistant mutations
Source: Commun Med (Lond). 2026 Mar 25;6:284. doi: 10.1038/s43856-026-01546-1 (PMC13181001; doi:10.1038/s43856-026-01546-1)
Supplement: Supplementary file 3 — Description of Additional Supplementary Files [file 43856_2026_1546_MOESM3_ESM.docx]

**Description of Additional Supplementary Files**

Supplementary Data 1: The coordinate Crystallographic Information File for 9KL4

Supplementary Data 2: The structure-factor Crystallographic Information File for 9KL4

Supplementary Data 3: The coordinate Crystallographic Information File for 9KLW

Supplementary Data 4: The structure-factor Crystallographic Information File for 9KLW

Supplementary Data 5: The source data for Figure 1b, 5a-i, 7a-c, 8b, 8c, and Table 1

Supplementary Data 6: The source data for Figure 2a and 2b

Supplementary Data 7: The source data for Figure 3a and 3b

Supplementary Data 8: The source data for Figure 4a and 4b
